# Gitelman syndrome, hypomagnesemia, and venous thrombosis: An intriguing association

**DOI:** 10.1002/ccr3.5542

**Published:** 2022-03-03

**Authors:** Soumaya Chargui, Rawnak Houli, Mondher Ounissi, Fethi Ben Hamida, Amel Harzallah, Ezzeddine Abderrahim

**Affiliations:** ^1^ Internal Medicine (A) Department Charles Nicolles Hospital Tunis Tunisia; ^2^ Research Laboratory of Renal Pathology LR00SP01 Charles Nicolles Hospital Tunis Tunisia

**Keywords:** Gitelman, heparin, hypomagnesemia, venous thrombosis

## Abstract

Among salt‐wasting tubulopathies' complications, venous thrombosis is one of the rarest. We report a case of a young woman with Gitelman syndrome (GS). She presented a deep venous thrombosis in her leg and was treated with heparin with favorable outcomes. We retained hypomagnesemia as the cause of the thrombosis.

## Graphical abstract

Gitelman syndrome's complications are mostly due to the metabolic disorders. Deep venous thrombosis is very rare complication in Gitelman syndrome. Deep venous thrombosis in Gitelman syndrome could be due to hypomagnesemia.

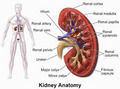

## 1 BACKGROUND

Gitelman syndrome (GS) is an autosomal recessive condition that results from defects in the sodium chloride cotransporter encoded by SLC12A3, resulting in primary salt‐wasting tubulopathy with hypokalemia, hypomagnesemia, hypocalciuria, and failure to thrive. Cardiac arrhythmia and seizures are the most dangerous complications due to these metabolic disorders.

Our work aims to report a much rarer complication that is deep venous thrombosis and discuss its pathogenesis and probable cause.

## 2 CASE PRESENTATION

A 33‐year‐old woman was admitted in December 2019 to the intensive care unit for a tetany crisis associated with severe hypokalemia. Neurological symptoms were improved after potassium supplementation. Physical examination was unremarkable once the tetany crisis was over. Blood pressure was 110/75 mmHg, and heart rate was 76 bpm. She had no volume overload. Preventive doses of heparin were given since admission (enoxaparin at the dose of 4000 IU per day). For the first 2 days of hospitalization, the patient suffered from muscle weakness and was resting for most of the day. On day 3, she began to stand and walk with help from the nurses. Two weeks after admission, she was transferred to the nephrology department. In her history, the patient had suffered from teenage years from fatigue, dizziness, constant thirst, and muscular cramps. The evaluation showed—in addition to hypokalemia—hypomagnesemia and metabolic alkalosis. Urine analysis showed renal potassium wasting, hypocalciuria, and polyuria. Further analysis was conducted showing negativity for antinuclear antibodies and anti‐Sjogren's syndrome A and B antigen antibodies. Laboratory findings in blood and urine at admission on our department are shown in Table 1. Immunological and endocrine findings are shown in Table 1. The diagnosis of GS was made. Genetic screening was unavailable in our country, and the patient did not have the means to have it sent abroad.

**TABLE 1:** Blood and urine analysis (day 14 at the hospital, upon transfer to the nephrology department)

Urine sodium	206 mmol/24 h (40–220)
Urine potassium	282 mmol/24 h (25–125)
Urine chloride	387 mmol/24 h (110–250)
Urine calcium	3.74 mmol/24 h (2.5–7.5)
Urine magnesium	25 mmol/24 h (2.4−6.5)
Serum creatinine	50 µmol/L
Sodium	141 mmol/L (range: 135–145)
Potassium	2.4 mmol/L (range: 3.5–5.5)
Chloride	99 mmol/L (range: 96–109)
Calcium	2.29 mmol/L (range: 2.2–2.6)
Magnesium	0.62 mmol/L (range: 0.85–1.1)
pH	7.49 (7.38–7.42)
Bicarbonate	24.2 mmol/L (range: 22–26)
Serum aldosterone	792.6 pmol/L (range: 137–487)
Active plasmatic renin	32.2 pg/ml (range: 3.18−32.6)
Antinuclear antibodies	Negative
Protein S	91 (range: 60–113)
Protein C	79 (range: 64–128)
Factor V Leiden mutation	Negative
Antithrombine III	103% (range: 80–120)
Anticardiolipin antibodies	Negative
Beta2 glycoprotein	Negative
Lupus anticoagulant	Negative

During her hospitalization, the patient presented with pain and edema in the right leg, an ultrasound was performed and showed deep thrombosis of the femoral vein, and the patient was treated with heparin and Acenocoumarol. No obvious thrombotic risk factors (such as obesity, prolonged bed rest, or chronic inflammation) were identified. The patient was not taking contraceptive treatment. There was no family history of venous thrombosis. Antiphospholipid antibodies were negative, and scanning for an inherited clotting blood disorder was also negative. However, magnesium levels were still low and IV supplementation was needed, alongside oral intake.

### 2.1 Outcome and follow‐up

The patient was discharged after 2 weeks and continued to receive oral potassium and magnesium supplementation. Acenocoumarol was maintained for 12 months. No recurrences of venous thrombosis were noted. The patient was admitted in November 2020 for severe hypokalemia with a favorable outcome. At the time, this article was written and she is still being followed at our clinics.

## 3 DISCUSSION

In 1966, Gitelman and colleagues described a salt‐wasting tubulopathy with severe hypokalemia, but that was different from the previously described Barter syndrome, since it associated renal wasting of magnesium.^1^ Since then, GS became more and more familiar and numerous cases were reported. It is also known as familial hypokalemia‐hypomagnesemia, due to its autosomal recessive transmission. GS is the consequence of a biallelic mutation affecting the SLC12A3 gene causing the inactivation of thiazide‐sensitive sodium chloride cotransporter (NCC) in the DCT,^2^ thus resulting in a salt‐wasting tubulopathy with volume contraction, hyper‐reninism, and hyper‐aldosteronism. This will cause increased sodium reabsorption in the collecting duct along with secretion of potassium and hydrogen ions. The mechanisms of magnesium wasting remain unclear, but it is believed to be a consequence of downregulation of the epithelial Mg channel, TRPM6.^3^ The final presentation will include hypokalemia, hypomagnesemia, hypocalciuria, and metabolic alkalosis. Symptoms often occur after the age of 6 but can manifest in adolescence or adulthood.^4^ Patient can present with fatigue, failure to thrive, muscular cramps, salt craving, and nocturia.^2^, ^4^ The diagnosis of GS is suspected on clinical manifestation and could be retained on biological findings following the criteria of the 2016 Kidney Disease Improving Global Outcomes (KDIGO) consensus. The gold standard is the genetic testing for SLC12A3 mutations; however, it is not accessible in all countries.

Gitelman syndrome complications are mostly due to the metabolic disorders associated with the disease, like cardiac arrythmia, prolonged QT, seizures, and rhabdomyolysis.^5^ Our case is particularly interesting since we report a very rare complication: deep venous thrombosis.

Venous thrombosis can be caused either by alterations in blood flow, endothelial vascular injury, or derangement in the constitution of blood. In our case, the patient had no underlying conditions causing blood thickening, and her blood circulation was not compromised by obesity or prolonged bed rest. Even though she had an ICU stay, she quickly resumed walking with assistance. This led us to suspect another factor could have a role in the pathogenesis of the thrombosis, that being the severe hypomagnesemia from which the patient suffered. In fact, the relationship between GS, hypomagnesemia, and vascular thrombosis has been studied in the previous work.^6^ Studies have shown that magnesium contributes to lowering oxidative stress and endothelial dysfunction^7^, ^8^, ^9^ and that low magnesium levels can result in a prothrombotic stage causing blood clots. However, most of the studies have dealt with magnesium effects on the arterial system, and there are very little data to whether the same mechanisms can be extrapolated to the venous system, thus causing deep venous thrombosis. Besides, even if these studies showed inverse correlation between magnesium levels and prothrombotic stage, further clinical proof remains needed to prove that GS patient with severe chronic hypomagnesemia do develop vascular thrombosis^9^, ^10^ and that is in fact why this case report is pertinent, since it could be, to our knowledge, the first case of deep venous thrombosis associated with hypomagnesemia in a GS's patient. To sum up, and in light of present data, we cannot affirm that hypomagnesemia alone caused this case of venous thrombosis, but evidence points that it could at least be an aggravating factor.

## 4 CONCLUSION

Gitelman syndrome is a rare and serious condition due to the severe metabolic disorders it induces, which can not only have neurological, cardiac, and muscular manifestations, but also thrombotic manifestations as shown in our patients' case. Further research for similar cases and the role of magnesium in this complication could be helpful for more understanding of this disease.

## CONFLICT OF INTEREST

The authors have no conflicts of interest to declare.

## AUTHOR CONTRIBUTIONS

RH involved in patient care and writing, SC involved in patient care and writing, MO involved in patient care, AH involved in patient care, EA involved in correction.

## ETHICAL APPROVAL

This work was done in accord with the World Medical Association Declaration of Helsinki.

## CONSENT

Written informed consent was obtained from the patient to publish this report in accordance with the journal's patient consent policy.

## Data Availability

All data underlying the results are available as part of the article and no additional source data are required.
